# Frequency and Nature of Incidental Extra-Enteric Lesions Found on Magnetic Resonance Enterography (MR-E) in Patients with Inflammatory Bowel Diseases (IBD)

**DOI:** 10.1371/journal.pone.0004863

**Published:** 2009-04-01

**Authors:** Hans H. Herfarth, Michael Grunert, Frank Klebl, Ulrike Strauch, Stefan Feuerbach, Jürgen Schölmerich, Gerhard Rogler, Andreas G. Schreyer

**Affiliations:** 1 Department of Internal Medicine, University of Regensburg, Regensburg, Germany; 2 Department of Radiology, University of Regensburg, Regensburg, Germany; Charité-Universitätsmedizin Berlin, Germany

## Abstract

**Background:**

The aim of this study was to determine the occurrence of extra-enteric findings in a large cohort of patients undergoing magnetic resonance enterography (MR-E) and to classify the clinical significance of these findings.

**Materials and Methods:**

We retrospectively analyzed 1154 MR-E performed in 1006 patients referred to our radiological department between 1999–2005. The reasons for referral were suspected or proven inflammatory bowel diseases (IBD) (n = 710), further diagnostic work-up for small bowel disease because of non-specific abdominal symptoms (SBD; n = 182) or suspected small bowel malignancies (SBM; n = 114). All extra-enteric findings were reviewed by a radiologist and a gastroenterologist and were classified as having high, moderate, or low significance for further diagnostic or therapeutic procedures.

**Results:**

The average age of all patients was 40±16 (Mean±SD) years (y) (IBD 35±13 y; SBD 49±16 y; SBM 57±15 y). A total of 1113 extra-enteric findings were detected in 600 of 1006 patients (59.6%). Of these findings 180 (16.2%) were judged as having a high, 212 (19.0%) a moderate and 721 (64.8%) a low significance. On a per group basis in patients with IBD 12.0% of the findings were of major clinical significance compared to 13.7% and 33.3% in patients with SBD and SBM, respectively. The most common major findings were abscesses (69.9%) in the IBD group and extraintestinal tumors, metastases or masses in the SBD and SBM groups (41.9% and 74.2%, respectively).

**Conclusions:**

MR-E reveals a substantial number of extra-enteric findings, supporting the role of a cross-sectional imaging method for the evaluation of the small bowel.

## Introduction

In patients with suspected or proven inflammatory bowel disease, radiological imaging techniques of the small bowel are employed to either establish or exclude the diagnosis of small bowel Crohn's disease or to evaluate the location, extent and the presence of strictures of small bowel in patients with established Crohn's disease. Until recently, the small bowel follow-through or the classical conventional enteroclysis have been the preferred radiological technique for the visualization of the small bowel. However, MR or CT based enterography or enteroclysis are more and more considered as a standard imaging procedure in patients with suspected or established inflammatory bowel diseases [1], [2], [3]. Especially the diagnostic value of MR imaging (MRI) of the small bowel in patients with inflammatory bowel disease has been extensively assessed in recent years using various contrast media and different techniques [4], [5], [6], [7], [8], [9], [10], [11], [12], [13], [14], [15]. Several studies also demonstrated a high correlation of this technique with conventional radiological methods such as small bowel follow-through or conventional enteroclysis, surgery and endoscopy [16], [17], [18], [19], [20], [21], [22], [23].

The advantage of both CT and MRI as compared to conventional radiological methods is the acquisition of additional information of extraluminal pathologies. Studies comparing MRI and conventional enteroclysis revealed additional pathological extra-enteric abnormalities in 25–58% of the cases, which are sometimes clinically relevant and lead to changes in medical or surgical management [18], [20], [24], [25], [26], [27]. All these studies have in common, that only small groups of patients were examined. Additionally, the clinical significance of these extra-enteric pathologies has never been evaluated.

Several studies analyzing the frequency and clinical significance of extracolonic findings in patients undergoing virtual colonography for screening purposes have been recently published. A systematic review of 17 studies including 3448 patients revealed that up to 40% of the patients had extracolonic pathologies [28]. In this analysis clinical significant findings necessitating further diagnostic or therapeutic work-up were described in 10.5% in a subgroup of 2787 patients. Since patients with IBD in general present at a younger age than patients undergoing screening colonography (age >50 years) and therefore most likely suffer from less comorbidity, we evaluated the prevalence and clinical significance of extraintestinal findings in this patient group. For this purpose we retrospectively analyzed all extra-enteric findings in patients referred to our institution for MR-E between 1999–2005 because of suspected or established IBD. We additionally included two other groups of patients undergoing MR-E for unexplained abdominal symptoms without clinical suspicion of inflammatory bowel disease and patients with suspected small bowel tumors. The aim of this study was to determine the frequency of extra-enteric findings in these three groups and to classify the clinical significance of these findings.

## Materials and Methods

We retrospectively analyzed the extra-enteric findings of 1154 MR-E performed in 1006 patients referred to our radiological department between 1999–2005. The institutional review board of the University of Regensburg approved the study. The reasons for referral were highly suspected (based on previous evaluations often including colonoscopy) or proven inflammatory bowel disease (IBD) (n = 710), further diagnostic work-up for a suspected small bowel disease (SBD) because of non-specific abdominal symptoms (n = 182) or suspected small bowel malignancies (SBM) (n = 114). All extra-enteric findings were reviewed by a radiologist and a gastroenterologist and were classified as having minor, moderate, or major significance.

Employing a previously published classification by Hellstrom et al., findings considered to be of no or little clinical importance were classified as minor and unlikely to require further diagnostic procedures or medical therapy [29]. Examples include e.g. small cystic liver lesions, renal cysts, small renal calcifications or degenerative changes of the spine. Findings of moderate clinical importance did not require immediate further diagnostic workup or therapy, but would likely to be verified later on either by chart review or by further clinical or radiologic follow up. Examples include adrenal masses of <2 cm in diameter, indeterminate cysts of various organs, gallstones, splenomegaly. Findings of definite clinical importance requiring immediate further diagnostic or therapeutic intervention (e.g. hydronephrosis, suspected tumors, aortal aneurysms, pleural effusions) were classified as being of major clinical importance.

### MR-E

All MRI examinations were performed using a 1.5T Scanner (Symphony; Siemens Medical Systems, Erlangen, Germany) and a circular polarized 4-element phased array body coil. For the abdominal MR-E performed between 1999–2001 the patient drank 2 l of pineapple juice (12.7 mg/l manganese-content) mixed with 20 mg methylcellulose within 2 hours for bright lumen MRE [15]. After 2002 the dark lumen technique [30] was utilized. For this technique the patients drank 2 liters of tap water during 1 hour continuously. To achieve sufficient small bowel distension we added 50 g mannitol and 5 g carob seed (Nestargel, Nestle, Munich, Germany) to 1-liter water. To reduce bowel peristalsis, patients without contraindications received 40 mg N-butyl-scopolamine intravenously (Buscopan; Boehringer, Ingelheim, Germany) in 100 mL 0.9% NaCl continuously during the examination as drip infusion. For the bright lumen as well as the dark lumen MRE the same MRI sequences were applied.

As a fast screener sequence a coronal true fast imaging with steady precession (TRUFI; TR/TE, 4.76/2.38 msec; flip angle, 60 degrees; slice thickness, 5 mm; 256 matrix; FOV, 450 mm) and an axial half-Fourier acquired single-shot turbo spin echo (HASTE; TR/TE, 1070/77 msec; flip angle, 150 degrees; slice thickness, 8 mm; 256 matrix; FOV, 400 mm) as a T2-weighted sequence were acquired. Before injecting contrast media intravenously, a T1-weighted 2D-FLASH sequence with axial orientation was performed, which was used as a baseline sequence for contrast uptake. Afterward, 0.2 mmol/kg body weight Gd-DTPA (Magnevist; Schering, Berlin, Germany) with a flow of 2 mL/sec was given intravenously followed after 70 seconds by a fat-suppressed axial 3D-FLASH sequence (TR/TE, 4.6/1.8 msec; flip angle, 25 degrees; slab thickness, 140 to 160 mm with 80 partitions; 512×210 matrix; FOV, 400 mm). Additionally a fat-suppressed axial and coronal T1-weighted 2D-FLASH was acquired. Scanner time for the whole examination was approximately 25 minutes (range, 21 to 29 min).

## Results

539 of the 1006 patients included in this study were females (54%). The youngest patient was 11 years and the oldest 90 years old. The average age ±SD in the patient group referred for IBD (n = 710), SBD (n = 182) and SBM (n = 114) were 35±13, 49±16 and 57±15 years, respectively.

### Radiological classification of extra-enteric pathologies in patients with IBD, SBD and SBM

Overall 1113 extra-enteric findings were detected in 600 of 1006 patients (59.6%). Of these 689, 226 and 198 extra-enteric findings were detected in 403 of 710 (56.8%), 119 of 182 (65.4%) and 78 of 114 (68.4%) patients with IBD, SBD and SBM, respectively (table 1).

**Table 1:** Clinical significance of extra-enteric findings in patients with inflammatory bowel disease (IBD), non-specific abdominal symptoms (SBD) and suspected small bowel malignancies (SBM).

Patients with findings (n)*	Total findings (n)	Clinical significance		
		Major n (%)	Moderate n (%)	Minor n (%)
IBD (403)	689	83 (12.1)	136 (19.7)	470 (68.2)
SBD (119)	226	31 (13.7)	39 (17.3)	156 (69.0)
SBM (78)	198	66 (33.3)	37 (18.7)	95 (48.0)

* several findings per patient possible.

### Extra-enteric findings of minor and moderate clinical significance

Overall 470, 156 and 95 findings of minor clinical significance were detected in patients with IBD, SBD and SBM, respectively. Most commonly ovarian-, kidney-and liver cysts were described in 8–25% of the patients (table 2). 136, 39 and 37 findings of moderate clinical significance were detected in patients with IBD, SBD and SBM, respectively. The most common findings were chlecysto- or choledocholithiasis, degenerative bone disease and lymphadenopathy, which was considered as not suspicious for intestinal lymphoma (table 3).

**Table 2:** Extra-enteric findings of magnetic resonance enterography (MR-E) with minor clinical significance in patients with inflammatory bowel diseases (IBD), non-specific abdominal symptoms (SBD) and suspected small bowel malignancies (SBM).

	IBD	n	% of all IBD patients*	SBD	n	% of all SBD patients*	SBM	n	% of all SBM patients*
Findings total (n)		470			156			95	
Most common findings	Ovarian cysts	110	15.5%	Kidney cysts	40	22.0%	Kidney cysts	29	25.4%
	Kidney cysts	67	9.4%	Ovarian cysts	19	10.4%	Liver cysts	11	9.6%
	Liver cysts	50	7.0%	Liver cysts	15	8.2%	Small amounts of ascites	6	5.3%
	Small amounts of ascites	50	7.0%	Small amounts of ascites	14	7.7%			
Findings of minor clinical significance in <5% of patients		243			68			49	

* several findings per patient possible.

**Table 3:** Extra-enteric findings of magnetic resonance enterography (MR-E) with moderate clinical significance in patients with inflammatory bowel diseases (IBD), non-specific abdominal symptoms (SBD) and suspected small bowel malignancies (SBM).

	IBD	n	% of all IBD patients[Table-fn nt103] *	SBD	n	% of all SBD patients[Table-fn nt103] *	SBM	n	% of all SBM patients[Table-fn nt103] *
Findings total (n)		136			39			37	
Most common findings	Lymphadenopathy#	50	7.0%	Cholecysto-or choledocholithiasis	6	3.3%	Cholecysto-or choledocholithiasis	6	5.3%
	Cholecysto-or choledocholithiasis	6	3.3%	Degenerative bone disorder	5	2.7%	Degenerative bone disorder	5	4.4%
	Degenerative bone disorder	14	2.0%	Uterus myomas	4	2.2%	Uterus myomas	4	3.5%
	Uterine myomas	12	1.7%	Splenomegaly	3	1.6%	Moderate amounts of ascites	4	3.5%
	Splenomegaly	7	1.0%	Lymphadenopathy#	3	1.6%	Slightly dilated kidney collecting tubules	3	2.6%
							Kidney hypoplasia	2	1.7%
							Splenomegaly	2	1.7%
							Abdominal wall herniation	2	1.7%
Findings of minor clinical significance in <1% of patients		47			18			9	

* several findings per patient possible.

* several findings per patient possible.

# considered not suspicious for lymphoma.

### Extra-enteric findings of major clinical significance

**Figure 1:**
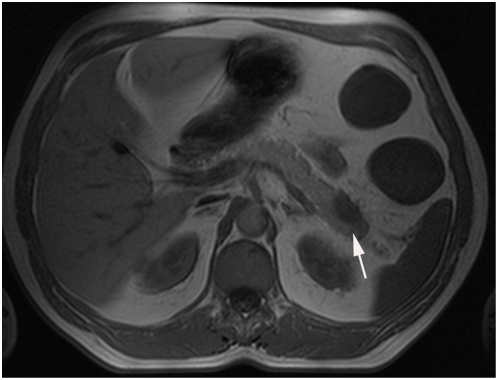
Pancreatic mass, which turned out to be pancreatic cancer in a 59-year old patient with Crohn's disease.

**Figure 2:**
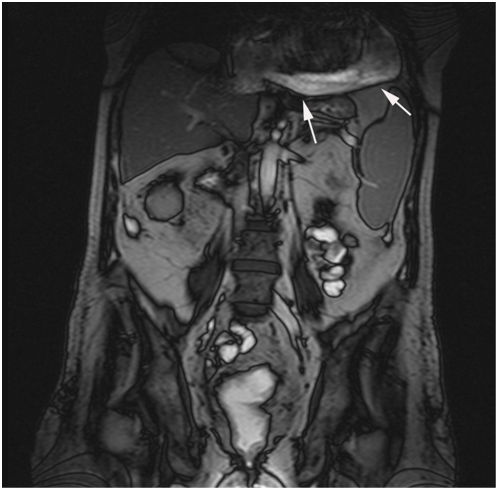
Pericardial effusion in a 47-year-old patient with active Crohn's disease.

Overall 180 findings were considered being of major clinical significance (see also table 1). The most common highly significant lesions were extraintestinal tumors (figure 1), metastases or masses (n = 72), abscesses (n = 63), pleural or pericardial effusions (n = 13) (figure 2) and hydronephrosis or ureteral obstruction (n = 16) (tables 4–6). 83 major clinical findings occurred in 73 of the 710 patients with IBD (10.3%) (table 4). In the group of SBD patient's 31 major clinical findings were detected in 27 of the 182 patients (14.8%) (table 5) and 66 major clinical findings were visualized in 40 of 114 SBM patients (35.1%) (table 6). The most common findings were extraintestinal abscesses in the IBD group (69.9% of all major clinical findings), whereas tumors, metastases or masses were most often described in the SBD and SBM group (41.9% and 74.2%, respectively). Of all patients with findings of major clinical significance 92.8% of the patients with IBD, 59.2% of the patients with SBD and 35.0% of the patients with SBM underwent further work-up. The work up was depending on the location of the findings and included abdominal or heart ultrasound, a conventional plain film of the thorax or bone structures.

**Table 4:** Major clinical extra-enteric findings in 73 patients with suspected or proven inflammatory bowel diseases (IBD) undergoing magnetic resonance enterography (MR-E) (n = 710).

Findings	n
Total	83
Abscess	58
Urethral obstruction	5
Pleural effusion	4
Extraintestinal mass*	2
Hydronephrosis	2
Lymphadenopathy, suspicion of lymphoma*	2
Bone metastasis*	2
Pericardial effusion	1
Pleural empyema	1
Tumor adrenal gland*	1
Solid pancreatic mass*	1
Solid ovary mass*	1
Bone tumor*	1
Bone necrosis	1
Bone fracture	1

* Classified as tumor, metastasis or mass; n = 10.

**Table 5 pone-0004863-t005:** Major clinical extra-enteric findings in 27 patients with non-specific abdominal symptoms (SBD) undergoing magnetic resonance enterography (MR-E) (n = 182).

Findings	n
Total	31
Abscess	6
Pleural effusion	5
Tumor adrenal gland*	4
Extraintestinal mass*	3
Urethral obstruction	2
Bone metastasis*	2
Hydrops of gallbladder	1
Aortic aneurysm	1
Celiac artery stenosis	1
Venous thrombosis	1
Pulmonary mass*	1
Renal mass*	1
Hydronephrosis	1
Pancreatic mass*	1
Bone tumor*	1

* Classified as tumor, metastasis or mass; n = 13.

**Table 6:** Major clinical extra-enteric findings in 40 patients with suspected small bowel malignancy (SBM) undergoing magnetic resonance enterography (MR-E) (n = 114).

Findings	n
Total	66
Extraintestinal mass*	16
Lymphadenopathy, suspicion of lymphoma*	16
Liver metastases*	8
Adnexal mass*	4
Pleural effusion	4
Aortal aneurysm	4
Urethral obstruction	4
Bone metastases*	2
Hydronephrosis	2
Abscess	1
Prostate cancer*	1
Mass rectus abdominis muscle*	1
Bone tumor*	1
Bone necrosis	1
Bone fracture	1

* Classified as tumor, metastasis or mass; n = 49.

Given the large percentage of patients with tumors, metastases or masses, a systematic medical chart review of these patients was performed. Nine patients in the IBD group presented with 10 extraintestinal findings classified as tumor, metastasis or mass. Five out of these 10 findings (50%) were newly diagnosed in 5 patients, whereas in 4 patients 5 extraintestinal masses were already known before the MRE examination by either a recently performed abdominal ultrasound examination or a CT-scan. In 12 patients with SBD and 33 patients with SBM 7 of 13 (53.8%) and 11 of 49 (22.4%) of the findings classified as tumor, metastasis or mass were newly detected.

## Discussion

MR-E is a newly evolving imaging technology to visualize the small bowel [31]. Aside of the lack of radiation, the advantage of this modality compared to the conventional radiological methods such as a small bowel follow through or conventional enteroclysis is the ability to detect extraintestinal pathology. In our retrospective study in nearly 60% of patients extra-enteric findings were detected. Whereas the majority of the findings were of low clinical importance, 35.2% were considered of high and moderate clinical significance. Extraintestinal findings of moderate importance were detected in 19.7%, 17.3% and 18.7% in the IBD, SBD and SBM groups. In the group of IBD and SBD patients 12.1% and 13.7% of all extraintestinal findings were considered of major clinical importance, whereas this was the case in 33.3% in the patients with suspected SBM.

Additional extra-enteric findings in 20–60% of the IBD patients are described in a number of published studies analyzing the efficacy of MR-E or CT-E in patients with IBD [18], [19], [20], [22], [24], [25], [26], [27], [32], [33], [34], [35]. However, in contrast to the previous studies, this study analyzed the clinical significance of extraintestinal findings in patients with IBD undergoing MR-E. The results demonstrate, that most often extra-enteric findings in patients with IBD are of minor or moderate clinical significance. Findings of major clinical importance were observed in approximately every tenth patient (73 of 710 patients with IBD). In the majority of the cases these were abscesses (58 of 83 major findings; 69.9%). Only 25 of the 83 major clinical findings were not abscesses (3.6% of all findings in IBD patients), which highlight this important complication in this patient group and indicates the value of this cross sectional method for further therapeutic decisions in the therapy of these patients, which is usually antibiotic therapy, CT or ultrasound-guided drainage and/or surgery [36]. The high number of patients presenting with abscesses could be also due to the fact, that the retrospective analysis was performed in a tertiary care center, which normally treats more complex IBD patients.

In 5 patients new extraintestinal findings were classified as highly suspicious of tumor, a metastasis or an abdominal mass. Even if this comprises less than 1% of the 710 IBD patients, given the generally young age of these patients, these findings have profound implications for the further clinical approach.

The large number of major extraintestinal findings in the SBM group in this retrospective study may be explained by the fact, that this patient group underwent the examination already with a high suspicion of an intestinal tumor, lymphoma or tumor metastases. Additionally only 11 of the 49 findings were new findings, whereas the majority had been previously diagnosed by another imaging technique. Overall the patients in this group were also in average 22 and 6 years older compared to the IBD and SBD group.

Several prospective and retrospective studies report between 20–70% of incidental extracolonic findings in patients undergoing virtual colonography for colorectal cancer screening. In this patient population the frequency of clinically important findings clearly depends on risk stratification. Whereas in average risk cohorts undergoing CT-colonography 4–6% of clinically important extracolonic findings are detected [37], the percentage of these findings increases to 10–23% in the so called high-risk population for colorectal cancer (defined as: family history of colorectal cancer, personal history of polyps, new onset of anemia) [29], [38], [39]. It is difficult to compare these findings with our results, since the patient cohorts in our study are clearly different from the patients studied in the colorectal cancer screening studies. All patient underwent the MR-E with a clinically based suspicion of small bowel disease, whereas in the colorectal cancer screening studies most of the patients (except the anemia patients) were not symptomatic.

A weakness of the presented study is it's retrospective character. This may especially affect the comparison of the IBD and SBD patient groups. We classified the patients according to the indication for the examination and not for the outcome. Therefore some patients in the SBD group may have been later classified as IBD and also some patients with suspected IBD may have been later classified with e.g. irritable bowel syndrome. However, since both groups had nearly similar prevalence rates in the 3 different categories of the extraintestinal findings, the overall result is most likely not affected. Retrospectively, it is also difficult to determine the further diagnostic follow-up based on the extraintestinal MR-E findings: often patients undergo several diagnostic procedures and it is difficult to determine, if new diagnostic procedures were ordered based on the MR-E findings or other clinical decisions.

In the setting of the retrospective character of the study we were also not able to determine, how many of the extraintestinal findings would have been detectable using other imaging modalities, especially abdominal ultrasound. Abdominal ultrasound is known to have a good sensitivity and specificity in detecting intestinal inflammation such as small bowel Crohn's disease, fistulizing disease or abscesses [40], [41], [42] Since abscesses are one of the main extraintestinal findings in patients with suspected or established Crohn's disease an abdominal ultrasound examination would be probably considerable cheaper and faster than an MRI exam, if such a complication is suspected. However; the sensitivity of abdominal ultrasound in detecting extraintestinal pathologies other than abscesses or fistulizing disease has never been determined in comparison e.g. with MRI or CT.

In conclusion, in more than half of the patients undergoing MR-E additional extra-enteric findings were detected, the majority of extra-enteric MR-E findings being of low clinical importance, but approximately one third of the patients being diagnosed with findings of moderate or major clinical significance. Especially in the group of IBD patients, in whom the majority of the described major clinical findings were abscesses, cross sectional imaging MR-E provides additional important clinical information compared to the conventional small bowel techniques such as SBFT or SB-enteroclysis.
